# Characterization of MenA (isoprenyl diphosphate:1,4-dihydroxy-2-naphthoate isoprenyltransferase) from *Mycobacterium tuberculosis*

**DOI:** 10.1371/journal.pone.0214958

**Published:** 2019-04-12

**Authors:** Rakesh K. Dhiman, Venugopal Pujari, James M. Kincaid, Melanie A. Ikeh, Tanya Parish, Dean C. Crick

**Affiliations:** 1 Department of Microbiology, Immunology and Pathology, Colorado State University, Fort Collins, CO, United States of America; 2 Queen Mary University of London, Barts & The London School of Medicine and Dentistry, London, United Kingdom; 3 TB Discovery Research, Infectious Disease Research Institute, Seattle, WA, United States of America; University of Padova, Medical School, ITALY

## Abstract

The menaquinone biosynthetic pathway presents a promising drug target against *Mycobacterium tuberculosis* and potentially other Gram-positive pathogens. In the present study, the essentiality, steady state kinetics of MenA from *M*. *tuberculosis* and the mechanism of MenA inhibition by Ro 48–8071 were characterized. MenA [isoprenyl diphosphate:1,4-dihydroxy-2-naphthoate (DHNA) isoprenyltransferase] catalyzes a critical reaction in menaquinone biosynthesis that involves the conversion of cytosolic DHNA, to membrane bound demethylmenaquinone by transferring a hydrophobic 45-carbon isoprenoid chain (in the case of mycobacteria) to the ring nucleus of DHNA. *Rv0534c* previously identified as the gene encoding MenA in *M*. *tuberculosis* complemented a *menA* deletion in *E*. *coli* and an *E*. *coli* host expressing *Rv0534c* exhibited an eight-fold increase in MenA specific activity over the control strain harboring empty vector under similar assay conditions. Expression of *Rv0534c* is essential for mycobacterial survival and the native enzyme from *M*. *tuberculosis* H37Rv was characterized using membrane preparations as it was not possible to solubilize and purify the recombinant enzyme. The enzyme is absolutely dependent on the presence of a divalent cation for optimal activity with Mg^+2^ being the most effective and is active over a wide pH range, with pH 8.5 being optimal. The apparent K_m_ values for DHNA and farnesyl diphosphate were found to be 8.2 and 4.3 μM, respectively. Ro 48–8071, a compound previously reported to inhibit mycobacterial MenA activity, is non-competitive with regard to DHNA and competitive with regard to the isoprenyldiphosphate substrate.

## Introduction

The electron transport chain (ETC) involved in oxidative phosphorylation, consists of dehydrogenases and terminal reductases that are linked by isoprenoid lipoquinones. The nature of the isoprenoid lipoquinones found in the ETC varies from organism to organism. Eukaryotic cells synthesize and utilize ubiquinone, a benzoquinone, in their ETC, whereas many prokaryotes, such as Gram-negative *Enterobacteriaceae* tend to contain ubiquinones, menaquinones (naphthaquinones, MK), demethylmenaquinones (DMK) or a combination of these compounds, and strictly aerobic Gram-negatives generally synthesize only ubiquinones. However, members of the Gram-positive taxa typically synthesize either MK, DMK or in the case of the *Actinomycetes* and Coryneform bacteria possessing mycolic acids, partially hydrogenated MK [1]. *Mycobacterium* spp. synthesize MK with isoprenoid chains of nine isoprene units, with one of these being saturated reviewed in [2] and is designated MK-9(H_2_) here. In general, the chain-length and degree of saturation of the isoprenoid moiety is used to identify these compounds; for example, menaquinone from *Escherichia coli* is designated MK-8 [menaquinone with eight isoprene units (40 carbon atoms)].

Since MK is a central component in the respiratory chain of mycobacteria, and humans do not utilize MK in their ETC, MK synthesis is receiving considerable interest as an attractive target for development of novel chemotherapeutics [3–9] to combat *M*. *tuberculosis* and potentially other pathogens that exclusively synthesize this compound.

**Fig 1:**
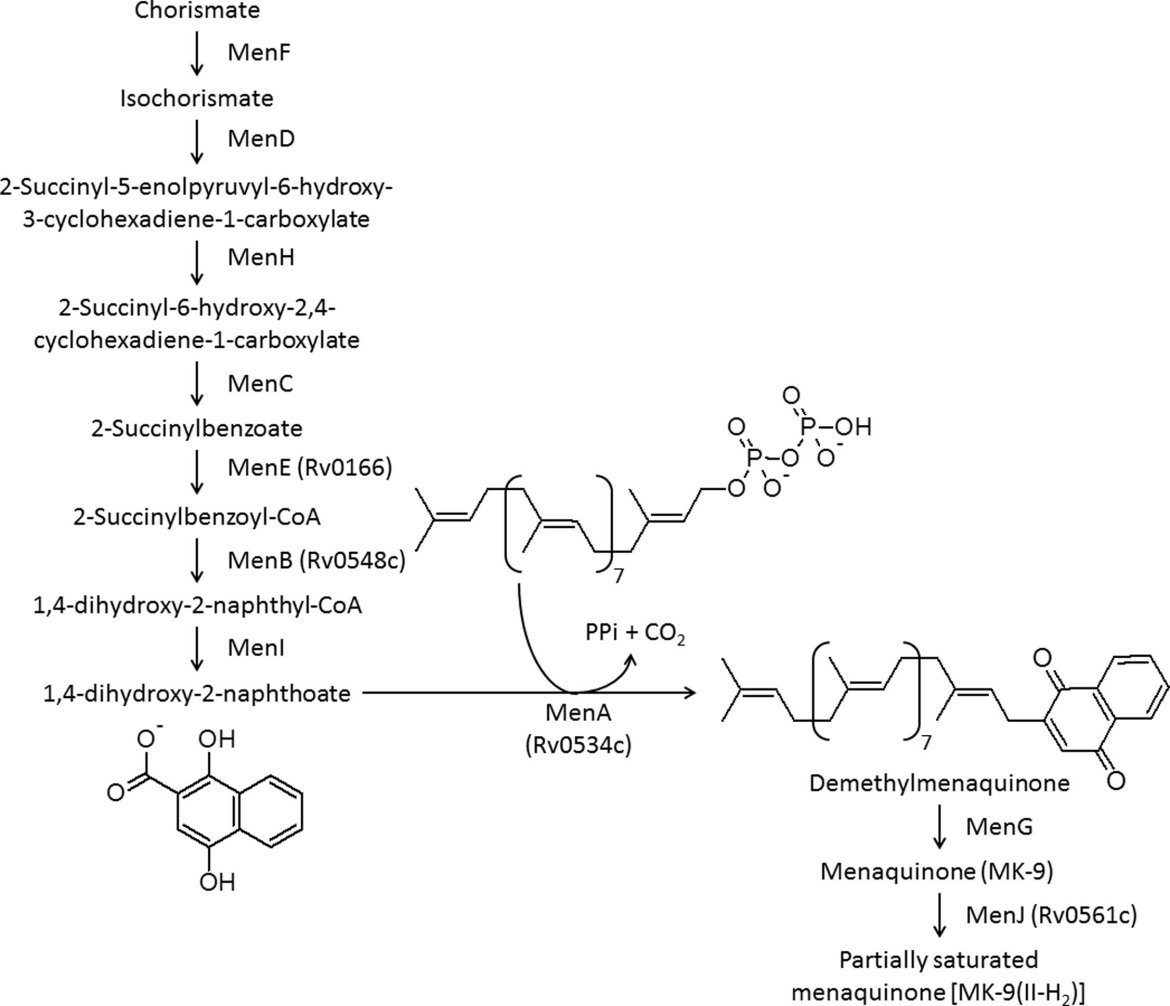
Biosynthetic pathway for menaquinone in mycobacteria. The reaction catalyzed by MenA is shown. Rv numbers have been provided for enzymes for which the activity has been empirically demonstrated.

MK biosynthesis in mycobacteria results from the convergence of the shikimate pathway and isopentenyl diphosphate synthesis, and is catalyzed by ten enzymes, MenA-MenJ [Fig 1 [10]]. MenA (isoprenyl diphosphate:1,4-dihydroxy-2-naphthoate (DHNA) isoprenyltransferase), an aromatic prenyltransferase [11,12], catalyzes a critical reaction in MK biosynthesis that involves the conversion of the cytosolic DHNA, to membrane bound DMK by transferring a hydrophobic 45-carbon isoprenoid chain (in the case of mycobacteria) to the position of the ring nucleus of DHNA previously occupied by the carboxylic acid function [Fig 1 [9,13,14]].

Although the gene encoding MenA in *Escherichia coli* was identified in 1976 [15] it was not cloned from any organism until 1998 [16]. MenA activity was demonstrated in *E*. *coli* [15] and subsequently *Micrococcus luteus* membranes [17]; however, MenA has yet to be purified, the reaction mechanism remains unclear and, surprisingly, the kinetic properties of this enzyme have not previously been described. Given the current interest in MK synthesis in general and our interest in MenA, specifically, as a target for chemotherapeutics we have determined the essentiality of *M*. *tuberculosis* MenA, characterized the enzyme and investigated the mechanism of inhibition by Ro 48–8071, a compound developed as an oxidosqualene cyclase inhibitor and previously shown to inhibit mycobacterial MenA activity at low micromolar concentrations [3].

## Materials and methods

### Bacterial strains and chemicals

Gamma-irradiated whole *M*. *tuberculosis* H37Rv cells and H37Rv genomic DNA were provided by BEI Resources. All PCR product and plasmid purifications were performed using Qiagen kits, antibiotics and DHNA were purchased from Sigma-Aldrich. Bio-Rex 70 cation exchange resin was obtained from Bio-Rad. [1-^3^H]Farnesyl diphosphate (FPP, 23 Ci/mmol), and [1-^3^H]geranylgeranyl diphosphate (GGPP, 17 Ci/mmol) were purchased from Perkin Elmer and Amersham Biosciences, respectively. All other chemicals and reagents were purchased from Sigma-Aldrich unless otherwise noted.

*E*. *coli* BL21 (DE3) was purchased from Novagen and *E*. *coli* wild type (CGSC # 7636) and an *E*. *coli menA* disruption mutant (CGSC # 10816) were procured from the *E*. *coli* Genetic Stock Center at Yale University [18]. Descriptions of bacterial strains, plasmids and primers can be found in S1 Table (Supporting Information).

### Media

Middlebrook 7H9 liquid medium was supplemented with 10% v/v OADC (Becton Dickinson) and 0.05% w/v Tween 80. Middlebrook 7H10 agar was supplemented with 10% v/v OADC. Hygromycin was used at 100 μg/mL, kanamycin at 20 μg/mL, gentamicin at 10 μg/mL, sucrose at 1% w/v, and X-gal at 50 μg/mL where required. The ARO supplement contained 40 μg/ml of L-tryptophan, 40 μg/ml of L-phenylalanine, 40 μg/ml of L-tyrosine, 250 μM of *p*-hydroxybenzoate, 250 μM of *p*-aminobenzoic acid and 250 μM of 2, 3-dihydroxybenzoate.

### Construction of deletion vectors

A suicide delivery plasmid (pTACK102G) was constructed and in-frame deletion of *menA* was carried out as previously described [19]. Primer pairs MenAF1/MenAR1 (Supporting Information) and MenAF2/MenAR2 were used to amplify the upstream and downstream regions and these were cloned into p2NIL as (*SmaI-EcoRI* and *PstI-EcoRV* fragments respectively); the marker cassette (*hyg*, *lacZ*, *sac*) from pGOAL19 was inserted as a *Pac* fragment [19]. The complementing vector (pTACK119) was constructed by amplifying *menA* using primer pair MenAC1/MenAC2 and was cloned into pAPA3 under the control of the Ag85a promoter [20].

### Testing for essentiality

The suicide vector was introduced into wild-type *M*. *tuberculosis* H37Rv (ATCC25618) by electroporation [21] and single cross-overs (SCOs) were isolated on 7H10 agar plates containing 10% v/v OADC, hygromycin, kanamycin and X-gal. One blue colony was selected to generate double cross-overs (DCOs) on sucrose, X-gal plates as previously described [19]. White colonies were screened for the presence of the wild-type or deletion allele by PCR. A merodiploid strain was constructed by transforming the SCO with pTACK119 and selecting on hygromycin, kanamycin and gentamicin. DCOs were isolated and screened by PCR. Recombinant strains were confirmed by Southern hybridization. Gene switching was carried out as described to replace the resident integrated vector [22]; briefly, DCO strains complemented with an integrated copy of *menA* (from *M*. *tuberculosis*) were transformed with an empty vector, pUC-Hyg-Int [23] and recombinants isolated on hygromycin to select for the incoming vector.

### Complementation of the *menA* deletion in *E*. *coli*

Based on the nucleotide sequence of the *Rv0534c*, the primer pair MenAF3/ MenAR3 (Supporting Information) containing *Nde*I and *Hind*III restriction sites was designed and synthesized (Invitrogen). *Rv0534c* was amplified from *M*. *tuberculosis* chromosomal DNA using a Perkin-Elmer GeneAmp 2400 PCR system and Expand High Fidelity PCR system (Roche Diagnostics Corporation). The PCR product was digested with appropriate enzymes and cloned into pVV16, a shuttle and expression vector [24] containing *E*. *coli* and mycobacteria origins of replication [25]; the promoter (*hsp60*) is functional in *E*. *coli* and mycobacteria [26]. Plasmids, designated pVV16:MenA, were purified, analyzed by restriction digestion and sequenced (Macromolecular Resource Facility, Colorado State University).

Hence, competent cells were prepared from the *E*. *coli* Δ*menA* mutant (CGSC # 10816) and transformed with *M*. *tuberculosis Rv0534c* expressed constitutively under the control of the *hsp60* promoter from the replicative plasmid pVV16:MenA, and grown overnight in Luria-Bertani (LB) broth supplemented with hygromycin (150 μg/ml) at 37°C. *E*. *coli* Δ*menA* mutant and wild type (CGSC # 7636) strains were also grown under similar conditions in LB broth supplemented with 50 μg/ml kanamycin when appropriate.

### Extraction and analysis of menaquinone containing lipids

Cells were harvested by centrifugation and washed with water. The cell pellets were extracted twice with 20 volumes of CHCl_3_/CH_3_OH (2:1, V/V) and the combined extracts were washed with 1/5 volume of water. The upper, aqueous phase was discarded and the lower, organic phase was evaporated to dryness under a gentle N_2_ stream. The lipids were redissolved in CHCl_3_ and applied to a silicic acid column pre-equilibrated in the same solvent. Neutral lipids were eluted with CHCl_3_, the solvent evaporated under N_2_, and redissolved in C_2_H_5_OH for subsequent liquid chromatography-mass spectral (LC/MS) analysis. LC/MS was performed using an atmospheric pressure photo-ionization source in the positive mode on an Agilent 6220 time-of-flight mass spectrometer. A Waters XBridge (C18, 2.1X150 mm, 3.5 μm particle size) column heated to 40°C was used with a binary solvent system and a flow rate of 0.3 ml/min for the separation of the menaquinones. Chromatographic separation was done using a gradient of 100% solvent A (methanol) to 50% solvent B (isopropyl alcohol). The drying gas temperature was 350°C, the vaporizer temperature was set at 300°C and the fragmentor voltage was set to 120 V. Mass spectra were acquired from *m*/*z* 110 to 1800 with a frequency of one scan/sec.

### Expression of *Rv0534c* in *E*. *coli* BL21 (DE3) cells

A PCR primer pair (MenAF4/MenAR4) containing *Nde*I and *Hind*III restriction sites was designed (Invitrogen) for cloning *Rv0534c* into an *E*. *coli* expression vector. *Rv0534c* was amplified from chromosomal DNA as described above and the PCR product was digested with the indicated enzymes and ligated into the multiple cloning sites of pET28a(+) (EMD Biosciences, Inc.). The resulting plasmid, designated pET:MenA was used to transform *E*. *coli* DH5α subcloning cells (Life Technologies) for propagation. The plasmid was subsequently purified, analyzed by restriction endonuclease digestion and sequenced (Macromolecular Resource Facility, Colorado State University). The *E*. *coli* expression host BL21 (DE3) was transformed with pET:MenA, and grown to mid log phase (OD_600 nm_ ~ 0.6) in LB broth supplemented with kanamycin (50 μg/ml). IPTG was added (1mM final concentration) and incubation was continued for 4 hr at 30°C. Cells were harvested and stored frozen at -80°C for further processing.

### Membrane and cytosol preparations from *M*. *tuberculosis* and *E*. *coli* cells

Frozen cells (8–10 g) were thawed on ice in buffer A [50 mM potassium phosphate (pH 8.0), 300 mM NaCl, 5.0 mM MgCl_2_, 5 mM β-mercaptoethanol and 10% glycerol] at 2 ml/g of cells and sonicated on ice (10 cycles of 60 sec on and 90 sec off for mycobacteria and 10 cycles of 20 sec on and 60 sec off for *E*. *coli*), using a Sanyo Soniprep 150 (Integrated Services, TCP Inc.). Cell debris was pelleted by centrifugation at 25,000 X g for 20 min and discarded. The supernatant was centrifuged at 100,000 X g for 60 min to generate a membrane enriched fraction (pellet) and a cytosolic (supernatant) fraction. Membrane fractions were washed three times by repeated suspension in buffer A followed by centrifugation at 100,000 X g. Protein concentrations were determined using a BCA protein assay kit (Pierce).

### Purification of Rv0534c

For purification attempts 25,000 X g supernatants or membrane enriched fractions from the *E*. *coli* BL21 (DE3) cells transformed with pET:MenA were resuspended in buffer B [50 mM potassium phosphate (pH 8.0), 200 mM NaCl, 1 mM MgCl_2_, 1 mM β-mercaptoethanol and 10% glycerol] containing detergents at various concentrations and rocked at 4°C for 1–24 hr. The mixtures were then centrifuged at 100,000 X g to remove particulate material and the supernatant was incubated with Talon Co^++^ immobilized metal affinity resin (Clonetech, Palo Alto, CA), which was pre-equilibrated with buffer B containing the appropriate concentration of detergent and rocked at 4°C for 20 min. The loaded slurry was then packed in a column and washed with five bed volumes of buffer B containing 10 mM imidazole and appropriate detergents. The column was eluted step wise with aliquots of buffer B containing appropriate detergent and 50, 100, 150 or 200 mM imidazole. Fractions were analyzed by SDS–PAGE and Western blot.

### MenA assays

MenA activity was assayed by modification of a previously described protocol [15]. Reaction mixtures typically consisted of 0.1 M Tris-HCl buffer (pH 8.0), 5 mM MgCl_2_, 2.5 mM dithiothreitol, 0.1% CHAPS, membrane protein preparations (1mg/ml) and [1-^3^H]FPP (the specific activity of the radiolabeled material was reduced 45 fold with unlabeled material to achieve the indicated concentrations in the figure legends) in a total volume of 0.1 ml. The reactions were started by the addition of DHNA (250 μM final concentration) unless otherwise indicated in ethanol/diethyl ether (2:1 V/V) or DMSO (the final concentration of the organic solvent was less than 2% in all cases), which provided greater reproducibility in the kinetic analyses. Reaction mixtures were incubated at 37°C for a period where the reaction was linear with time, typically 30 min. Reactions were stopped by the addition of 500 μl of 0.1 M acetic acid in methanol and extracted twice with 4 ml of hexane. The hexane extracts were combined, evaporated to dryness under a stream of nitrogen and dissolved in CHCl_3_/CH_3_OH (2:1, V/V). An aliquot of the sample was used for scintillation spectrometry as described below and the remaining sample was loaded on C_18_ reverse phase thin layer plates, which were developed in acetone/water (95:5, V/V). Radioactive products were located on the thin layer plates using a Bioscan system 200 Imaging scanner and the proportion of total radioactivity co-migrating with unlabeled standards was used to calculate the total synthesis of DMK. For kinetic analyses samples were redissolved in diethylether/hexanes (5:95, V/V) and 0.2 g of silica was packed into a Pasteur pipette in the same solvent. The sample was loaded on the column in a minimal volume of solvent and the radiolabeled DMK was eluted with 3 ml of the solvent. The eluate was transferred to a scintillation vial, the solvent evaporated under a stream of nitrogen, 5 ml of Ultima Gold scintillation fluid (Perkin Elmer) was added and the radioactivity quantitated by liquid scintillation spectrometry on a Beckman LS 6500. In initial experiments the purity of the eluted [^3^H]DMK was verified by TLC as described above.

In some cases endogenous divalent cations were removed from the membrane preparations by cation exchange using Bio-Rex 70 resin. Optimal concentrations for divalent cations were then determined using the *in vitro* assay conditions described above with the indicated concentration of divalent cation. Optimal pH was determined using three buffering systems, 50 mM MES (pH 5.5–7.0), 50 mM TAPS (pH 7.0–9.0) or 50 mM CAPS (pH 9.0–10.5) and appropriate counter-ions to cover a broad pH range in increments of 0.5 pH units.

### Other procedures

BLAST searches were done on the National Center for Biotechnology Information website and the *Mycobacterium tuberculosis* Structural Genomics Consortium website using standard protein-protein BLAST (blastp). Alignments were done using multiple sequence alignments with hierarchical clustering using the ‘Multalin’ interface at the Institut National de la Recherche Agronomique website [27]. Restriction digests, ligations and electroporation were carried out as described [28] unless otherwise noted.

## Results

### Identification of a putative *menA* gene in *M*. *tuberculosis*

The *M*. *tuberculosis* genome contains a single open reading frame (*Rv0534c*) encoding a protein with similarity to MenA from *E*. *coli*. This gene, located near other genes annotated as being involved in menaquinone synthesis on the chromosome, was confirmed to encode functional MenA **[10]**. The protein encoded by *Rv0534c* was not predicted to be essential by high density transposon mutagenesis **[29]** but subsequent high-resolution phenotypic profiling suggested that the enzyme is essential for bacterial growth **[30]**. Rudimentary topological analysis with prediction programs such as TMHMM (DTU Bioinformatics website) suggest that MenA possesses seven transmembrane α-helices. A predicted ortholog of *Rv0534c* is retained in all *Mycobacterium* spp. queried including *M*. *leprae* and amino acid sequence alignments of predicted MenA proteins from different *Mycobacterium* spp. revealed greater than 80% identities. MenA from *E*. *coli* K12 has 33% identities and 495 positives when aligned with Rv0534c. Interestingly, Rv0534c also has 33% identities and 49% positives when aligned with UbiAD1 from *Homo sapiens*, which is reported to be a menaquinone biosynthetic enzyme widely distributed in human tissues **[31]**.

### MenA is essential *in vitro*

In attempts to delete *menA* in *M*. *tuberculosis* a suicide vector (pTACK102G) carrying an in-frame deletion of *menA* was constructed and used in a two-step homologous recombination procedure to isolate DCOs [19]. In the wild-type background no deletion mutants were obtained (0/40 tested). A merodiploid strain was constructed, by transforming the SCO with pTACK119, in which a copy of *menA* was integrated into the chromosome (using the L5 phage integration system) under the control of the Ag85a promoter. In this background, we were able to isolate DCOs with the deletion allele (21/40 recombinants); three strains were confirmed by Southern hybridization. These data suggest that the gene is essential under normal *in vitro* culture conditions and attempts to isolate *menA* deletion strains by supplementation with various components, including menaquinone, salicylate, aromatic amino acids, *p*-hydroxybenzoate, *p*-aminobenzoic acid, 2, 3-dihydroxybenzoate and methionine failed. Essentiality was tested by attempting to remove the integrated copy by gene switching [22]; no viable clones were isolated, confirming that *menA* is essential *in vitro*.

### MenA activity is associated with membranes

Cytosolic and membrane fractions from irradiated cells of *M*. *tuberculosis* H37Rv were initially assayed for MenA activity. Approximately 90% of the enzymatic activity seen in homogenates was associated with the membrane enriched fraction (while the remaining activity was localized to the 25,000 X g pellet and cytosol) when reaction mixtures contained either [1-^3^H]FPP or [1-^3^H]GGPP as the isoprenyl diphosphate substrate. Activities seen using the two allylic isoprenyl diphosphate substrates were similar. Assay mixtures containing [1-^3^H]geranyl diphosphate did not yield radiolabelled demethylmenaquinone as a product. Similarly, 4-hydroxybenzoic acid or menadione could not be substituted for DHNA as an isoprenyl acceptor.

### Detergent sensitivity of MenA activity

Since topology predictions indicated that the enzyme was likely membrane associated and the preliminary assays confirmed this prediction it was anticipated that there would be problems associated with protein expression and purification. Therefore, various detergents representing non-ionic, zwitterionic, anionic, and non-detergent sulfobetaines (NDSBs) were screened for the ability to support or enhance enzymatic activity in *M*. *tuberculosis* membranes and potentially facilitate solubilization of MenA for purification purposes. Many detergents, including Brij-35, CHAPS, Lubrol, Tween 80 and a NDSB, supported activity at a level >50% of the no detergent control while sodium deoxycholate, N-octylglucoside and NP-40 were clearly inhibitory when included in the assay mixture at 0.5% final concentration in an initial screen. Brij-35, CHAPS and Tween 80 were screened over a range of concentrations up to 4% of the reaction mixture to determine optimal and maximal concentrations that could be used. Both Brij-35 and Tween 80 supported enzymatic activity at levels several fold higher than the critical micelle concentration for these detergents. Although CHAPS stimulated activity at low concentrations this detergent inhibited the activity at or above 1% of the reaction mixture (S1 Fig).

### Expression and characterization of Rv0534c

**Fig 2:**
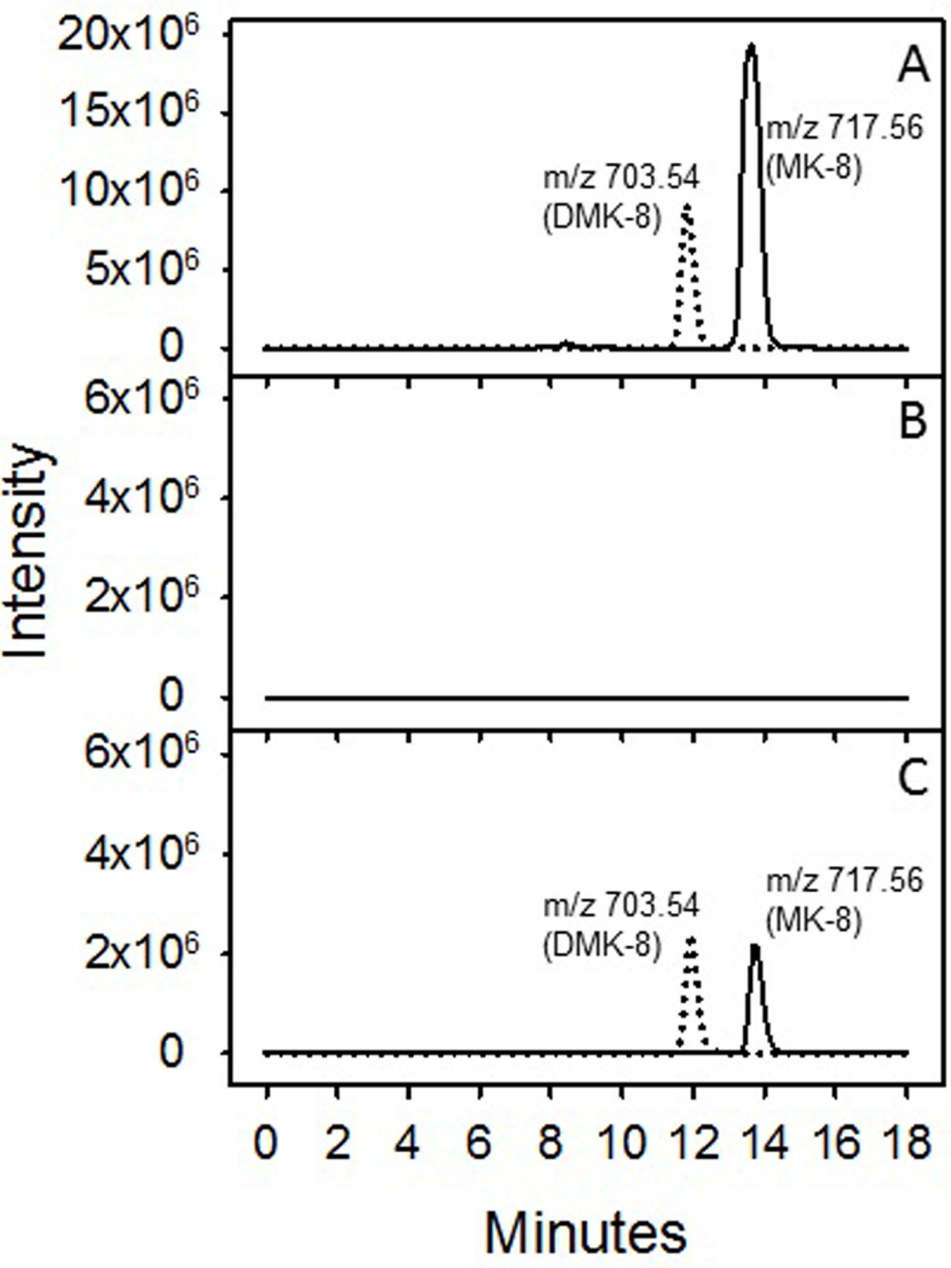
LC/MS analysis of neutral lipids. *E*. *coli* WT (CGSC # 7636) (Panel A), *E*. *coli* Δ*menA* (CGSC # 10816) (Panel B) and *E*. *coli* Δ*menA* (CGSC # 10816) complemented with pVV16:MenA (Panel C). Data shown are extracted ion chromatograms for *m/z* values of 703.54 (dotted line) and 717.56 (solid line), DMK-8 and MK-8, respectively.

LC/MS analysis of the neutral lipids derived from WT *E*. *coli* (CGSC # 7636) with an intact *menA* gene demonstrated that this strain synthesizes both MK-8 and DMK-8. Molecular features with m/z values of 717.56 and 703.54, corresponding to [M+H]^+^ ions of MK-8 and DMK-8, were prominent in the extracted ion chromatograms generated from this analysis (Fig 2, Panel A). The m/z value of each feature deviated from the predicted values by less than 2 ppm. However, the neutral lipids from the *E*. *coli* Δ*menA* mutant (CGSC # 10816) did not contain any detectable MK-8 or DMK-8 (Fig 2, Panel B), whereas synthesis of these compounds was restored in the Δ*menA* mutant (CGSC # 10816) transformed with pVV16:MenA (Fig 2, Panel C).

**Fig 3:**
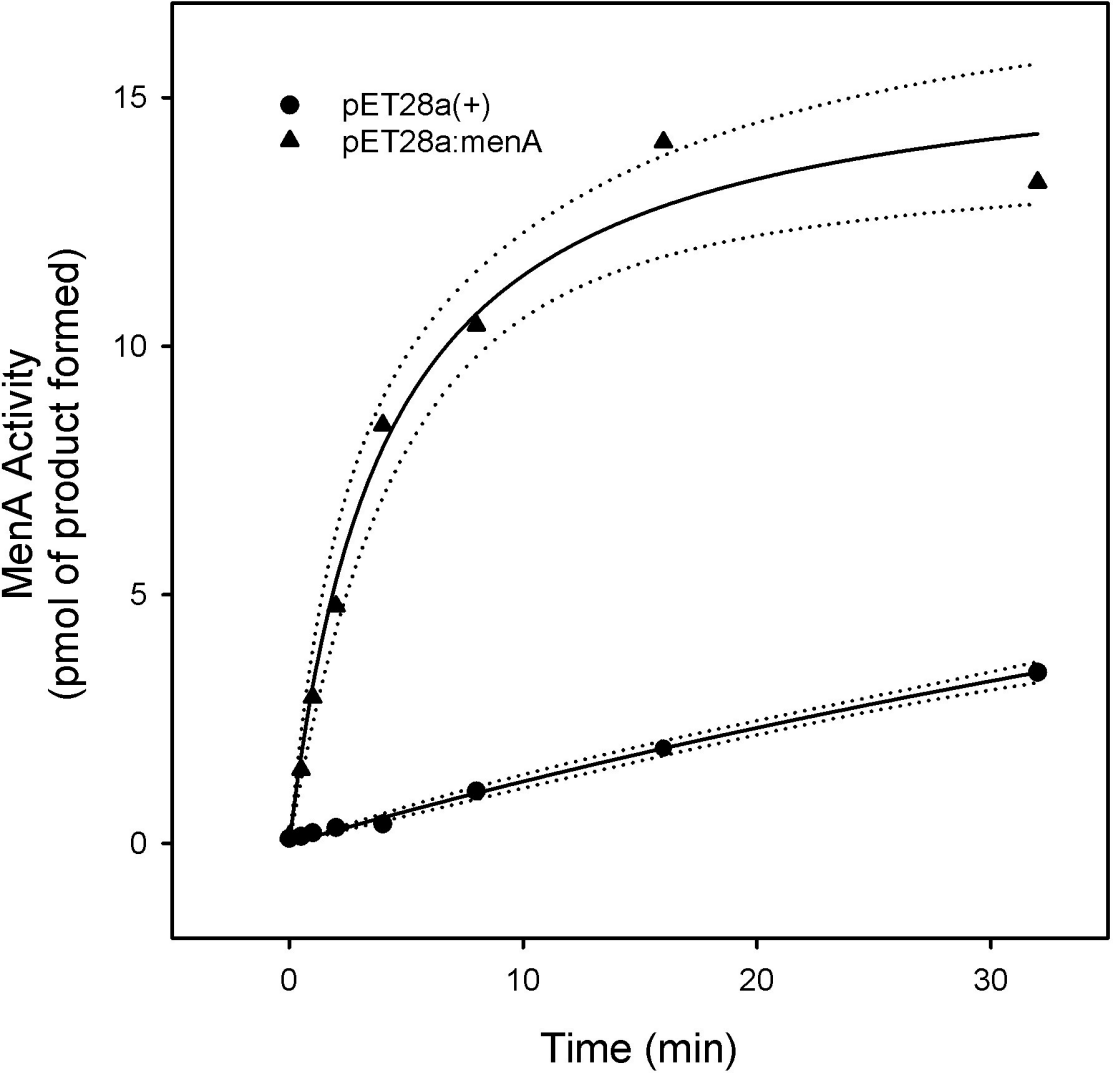
MenA activity in *E*. *coli* membranes. Membranes were prepared from *E*. *coli* BL21 (DE3) transformed with pET:MenA (open circles) or pET28a(+) (filled circles) grown to mid log phase. Reaction mixtures contained 0.1 M Tris-HCl buffer (pH 8.0), 5 mM MgCl_2_, 2.5 mM dithiothreitol, 0.1% CHAPS, membrane protein preparation (100 μg), DHNA (500 μM) and [1-^3^H]farnesyl diphosphate (10 μM). Mixtures were incubated at 37°C for 30 min and processed as described in Materials and Methods section. Data was fit to a single rectangular, 2 parameter hyperbola using SigmaPlot 13, the regression lines (solid) and the 95% confidence intervals (dotted) are shown. Statistical analysis can be found in Supporting Information.

To confirm that *Rv0534c* was responsible for encoding a protein with MenA activity the gene was also cloned into the expression vector pET28a(+) and used to transform *E*. *coli* BL21 (DE3) cells. Western blots, using an anti-polyHis antibody, of total cellular protein indicated that MenA was being expressed from the pET:MenA construct but subcellular fractionation indicated that the vast majority of the protein was in inclusion bodies and only small amounts of recombinant protein could be identified in the membrane preparations. Membrane preparations from *E*. *coli* harboring pET:MenA, or empty vector were used as enzyme sources for activity determinations. MenA specific activity was significantly greater (at least ~8 fold) in membranes isolated from the *E*. *coli* harboring pET:MenA than in membranes prepared from bacteria harboring the empty vector (Fig 3).

In attempts to solubilize the recombinant protein from *E*. *coli* BL21 (DE3) transformed with pET:MenA for purification purposes cells were sonicated and a 25,000 X g supernatant (containing the membranes and cytosol) or membrane enriched fraction was incubated at 4°C with various concentrations of detergents tested earlier (S1 Fig) followed by ultracentrifugation at 100,000 X g to remove particulate matter. However, enzyme assays of the resulting pellets and supernatants indicated that the activity remained in the pellet with no significant solubilization; in addition, SDS-PAGE and Western blot analysis suggested that there was significant aggregation of the recombinant protein in the particulates. Thus, as purification of the recombinant MenA was not possible, the native protein in membrane enriched preparations from *M*. *tuberculosis* was characterized.

### Catalytic properties and kinetics of inhibition

**Fig 4:**
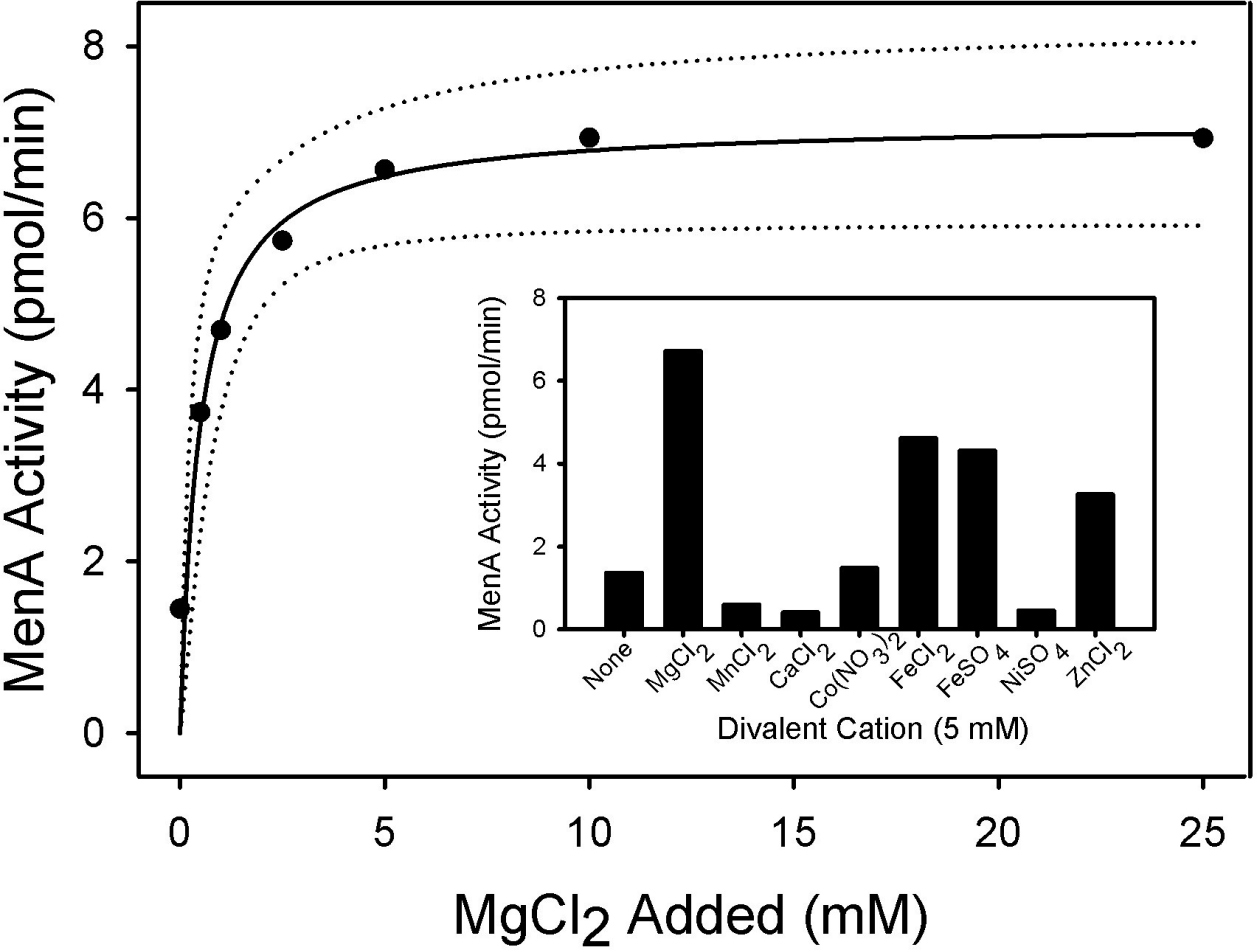
Effect of MgCl_2_ concentration on MenA activity. Reaction mixtures contained 0.1 M Tris-HCl buffer (pH 8.0), 2.5 mM dithiothreitol, 0.1% CHAPS, membrane protein preparation (100 μg) from *M*. *tuberculosis*, DHNA (500 μM), [1-^3^H]farnesyl diphosphate (10 μM) and MgCl_2_ at the indicated concentration. Mixtures were incubated at 37°C for 30 min and processed as described in Materials and Methods section. The inset shows the effect of various divalent cations on MenA activity when added to the reaction mixture at a final concentration of 5 mM. In all cases endogenous divalent cations were removed with Bio-Rex 70. Data was fit to a single rectangular, 2 parameter hyperbola using SigmaPlot 13, the regression line (solid) and the 95% confidence intervals (dotted) are shown. Statistical analysis can be found in Supporting Information.

**Fig 5:**
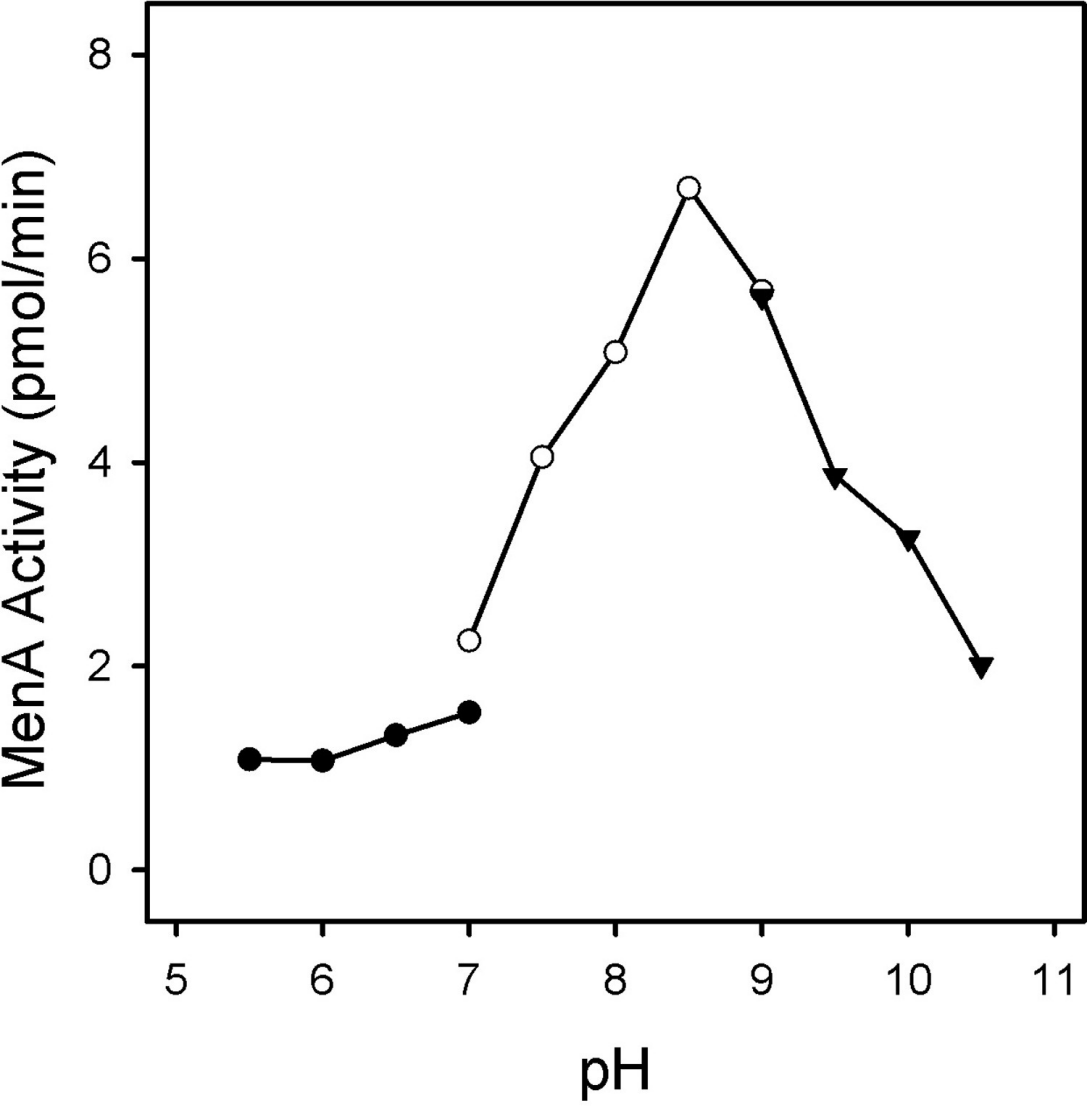
Effect of pH on MenA activity. Reaction mixtures contained 50 mM buffer (at the indicated pH), 5 mM MgCl_2_, 2.5 mM dithiothreitol, 0.1% CHAPS, membrane protein preparation (100 μg), DHNA (500 μM) and [1-^3^H]farnesyl diphosphate (10 μM). Mixtures were incubated at 37°C for 30 min and processed as described in Materials and Methods section. Three buffers, adjusted to the indicated pH with appropriate counter ions, were used: 50 mM MES (filled circles), 50 mM TAPS (open circles) or 50 mM CAPS (filled triangles).

MenA activity in membrane fractions from *M*. *tuberculosis* is absolutely dependent on the presence of divalent cations; the addition of 10 mM EDTA or stripping divalent cations from the membrane preparation with Bio-Rex 70 completely abolished enzymatic activity. Mg^2+^ ions supported maximal activity at concentrations above 5 mM (Fig 4). Fe^2+^ or Zn^2+^ ions also supported activity although at levels 50% lower than Mg^2+^ ions. The enzyme was nearly inactive in the presence of Mn^2+^, Ca^2+^ or Ni^2+^ ions. The enzyme was active over a broad range of pH values (7.0–10.5), with maximal activity at pH 8.5 (Fig 5). MenA activity in *M*. *tuberculosis* membranes was dependent on the addition of both FPP and DHNA to the reaction mixtures and apparent kinetic constants were determined using optimized assay conditions. The apparent K_m_ values for FPP and DHNA were found to be 4.3 and 8.2 μM, respectively. No substrate inhibition was observed with DHNA concentrations as high as 1 mM or FPP concentrations up to 65 μM.

**Fig 6:**
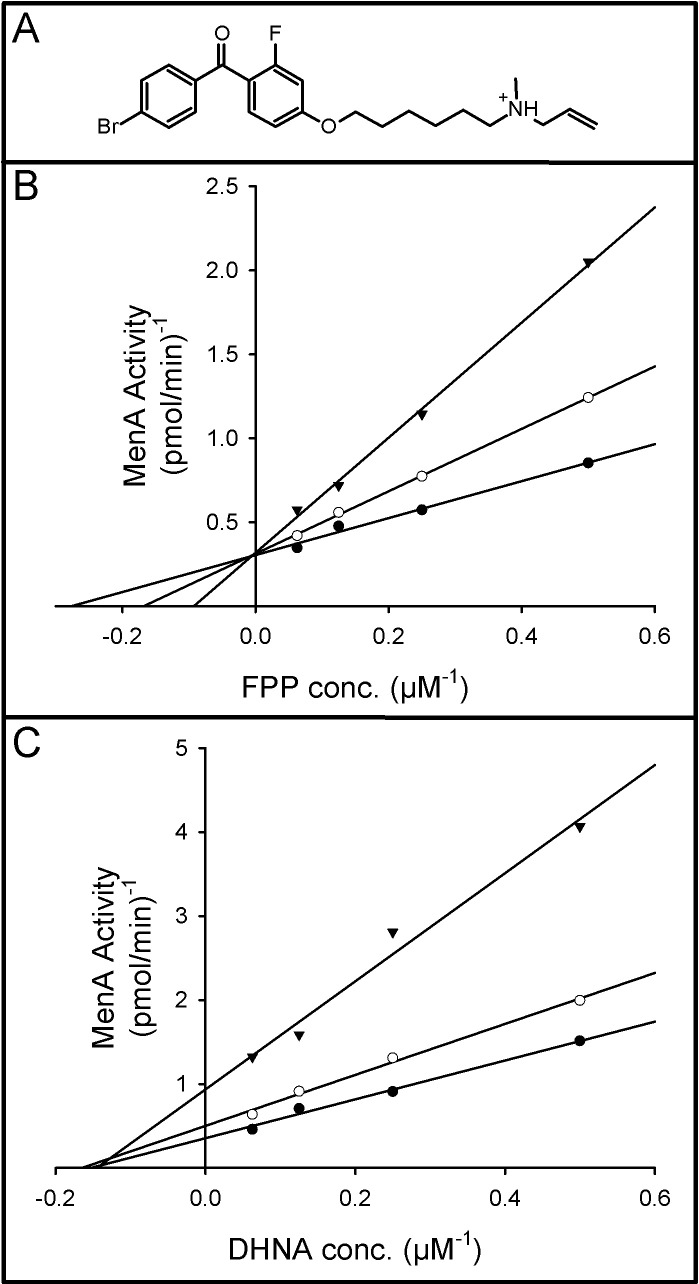
Effect of Ro 48–8071 on MenA activity. Double reciprocal plots showing the effect of Ro 48–8071 (structure Panel A) on the reaction rate at the indicated concentrations of [1-^3^H]farnesyl diphosphate and 250 μM DHNA (Panel B) or the indicated concentrations of DHNA and 20 μM [1-^3^H]farnesyl diphosphate (Panel C). In each case Ro 48–8071 was added at 0 (closed circles), 3 (open circles) or 7.5 (closed triangles) μg/ml. Reaction mixtures also contained 0.1 M Tris-HCl buffer (pH 8.0), 5 mM MgCl_2,_ 2.5 mM dithiothreitol, 0.1% CHAPS and membrane protein preparation (90 μg) from *M*. *tuberculosis*, in a final volume of 0.1 ml. Mixtures were incubated at 37°C for 30 min and processed as described in Materials and Methods section. Linear regression lines were generated using SigmaPlot 13. Regression analysis resulted in r^2^ values greater than 0.98 for all lines.

Fig 6 *s*hows the effect of Ro 48–8071 on MenA activity. The data indicates that Ro 48–8071 is competitive with respect to the allylic prenyl donor (FPP in this case). Whereas, Fig 6C indicates that Ro 48–8071 is noncompetitive with respect to DHNA.

## Discussion

MenA belongs to the UbiA family of prenyltransferases (IPR000537), which includes enzymes such as bacterial 4-hydroxybenzoate octaprenyltransferases (UbiA); yeast mitochondrial para-hydroxybenzoate-polyprenyltransferase (COQ2); and protohaem IX farnesyltransferase (haem O synthase) from yeast and mammals (COX10), and from bacteria (CyoE or CtaB) (EMBL-EBI website). It should be noted that in corynebacteria and mycobacteria the name UbiA was accepted for decaprenyl-phosphoryl-5-phosphoribose synthase [32], which is a member of the UbiA superfamily [12], but is not a canonical UbiA (4-hydroxybenzoate prenyltransferase).

A MenA homolog has yet to be purified. However, homology modeling techniques, secondary structure prediction tools, molecular dynamics simulations, and energy optimizations were used to generate a model of UbiA with two potential active sites [33]. Subsequently, the crystal structures of archaeal UbiA homologs from *Aeropyrum pernix* (ApUBiA) [34] and *Archaeoglobus fulgidus* (AfUbiA) [35] confirmed the active site prediction. The overall folds of the two structures were similar with nine transmembrane helices containing two Asp-rich motifs, NDXXDXXXD and DXXXD (N-Asn, D-Asp and X-any amino acid), and another conserved motif, YXXXK (Y- Tyr, X- any amino acid and K-Lys). These three motifs are also present in MenA from *M*. *tuberculosis* (S2 Fig).

The sequence similarity between UbiA and MenA suggests that the two enzymes likely have similar overall structures. Both of these enzymes catalyze the prenylation of an aromatic ring structure to form a benzoquinone in the case of UbiA and a naphthoquinone in the case of MenA. However the reaction catalyzed differs between these enzymes. In ubiquinone synthesis the carboxylic function is removed from the 4-hydroxybenzoate after alkylation by UbiA, whereas in MK synthesis the alkylation by MenA appears to involve alkylation and decarboxylation in a single step. In isotopic tracer experiments using whole cells it was determined that DHNA prenylation occurs at the position previously occupied by the carboxylic acid moiety [14,36]. This difference in reaction is likely reflected in the observation that sequence clustering analysis places UbiA and MenA homologs in separate clusters [12].

As indicated in the Results, Rv0534c also aligns with the human UbiAD1, which is reported to be a MK [31] or a ubiquinone [37] biosynthetic enzyme. If UbiAD1 is involved in MK synthesis, the reaction catalyzed is very different from that of either bacterial UbiA or MenA but sequence clustering analysis places UbiAD1in the same cluster as MenA [12]. UbiAD1 is reported to alkylate menadione or cleave the side-chain from Vitamin K1 (phylloquinone) or vitamin K2 (MK with a side-chain of 4 isoprene units) and realkylate the resulting menadione, using geranylgeranyl diphosphate as a substrate, generating vitamin K2 [31]. However, if UbiAD1 is involved in ubiquinone synthesis it utilizes 4-hydroxybenzoate as a substrate [37]. Mycobacterial MenA, Rv0534c, does utilize geranylgeranyl diphosphate as an alkylating substrate *in vitro* but preferentially utilizes solanesyl diphosphate (9 isoprene units) for that purpose *in vivo*. Rv0534c does not utilize menadione, Vitamin K or 4-hydroxybenzoate as a substrate suggesting that there is little similarity between the reactions catalyzed of Rv0534c and UbiAD1 and that it is unlikely that inhibitors of one would inhibit the other.

The results presented here represent the first detailed characterization of an enzyme activity that was first demonstrated many years ago, is poorly understood and which has recently generated interest as a drug target in *M*. *tuberculosis* and, potentially, other Gram-positive pathogens. Open reading frame *Rv0534c* from *M*. *tuberculosis* H37Rv genome encodes an isoprenyl diphosphate:DHNA isoprenyltransferase generating DMK, the precursor of MK, as this gene complements the defect in the MenA deficient *E*. *coli* strain (CGSC # 10816) and expression of *Rv0534c* in a heterologous host resulted in an 8-fold increase in the specific activity of MenA in membranes isolated from that organism. Highly conserved orthologs of this gene are present in all pathogenic *Mycobacterium* spp. investigated including *M*. *leprae*. The chromosomal localization and organization of the putative menaquinone biosynthetic gene cluster is also well conserved among mycobacterial spp., suggesting conservation of function.

Although recombinant enzyme was expressed in an active form in *E*. *coli* cells the protein proved to be refractory to purification. This is in keeping with the fact that, to our knowledge, no homolog of MenA has yet been purified and the suggestion that there may be only a small number of proteins in the UbiA superfamily that behave well enough to be pursued for crystallographic studies [12]. The *M*. *tuberculosis* enzyme is membrane bound as predicted by the TMHMM server at the Center for Biological Sequence Analysis, Technical University of Denmark and reported for the orthologs in *E*. *coli* and *M*. *luteus* [15,17]. Interestingly, although many detergents supported MenA activity *in vitro*, only one, CHAPS, stimulated the activity and then only very modestly. This is in contrast to the earlier reports that indicated that Triton X-100 is required for the activity of *E*. *coli* MenA [15] and that the enzyme from *M*. *luteus* was inhibited by the addition of detergents to reaction mixtures [17]. However, it should be noted that NP-40 (a detergent structurally similar Triton X-100, reported to inhibit the *M*. *luteus* enzyme) inhibited the *M*. *tuberculosis* enzyme.

Both enzyme assay conditions and product workup were optimized prior to kinetic analysis. Dissolving the DHNA in DMSO for addition to the reaction mixtures and developing facile column purification for synthesized DMK proved critical for reducing variability in the kinetic analyses. The enzyme is active over a broad range of pH and was maximal at pH 8.5. The activity was absolutely dependent on the presence of divalent cations, as addition of EDTA to the reaction mixtures or removal of cations by ion exchange chromatography abolished activity, a result consistent with the known catalytic mechanisms of aromatic prenyltransferases [11] and the archaeal UbiA crystal structures [34,35]. It was determined that the activity was optimal in the presence of Mg^2+^ ions at concentrations of 5 mM or more, which is also consistent with previous reports [15,17]. Other divalent cations also supported activity but the enzyme was relatively inactive in the presence of Mn^2+^ or Ca^2+^. The addition of dithiothreitol or β-mercaptoethanol to the reaction mixtures did not affect the activity.

MenA from *M*. *tuberculosis* has a strong affinity for DHNA with a calculated apparent K_m_ of 8.2 μM (all kinetic constants reported here are apparent as the assays were conducted in membrane preparations where the precise concentration of enzyme was unknown). MenA from *M*. *tuberculosis* was incapable of prenylating either menadione or 4-hydroxybenzoate showing absolute specificity for DHNA. This specificity appears to be a property of all isoprenyltransferases that prenylate aromatic ring structures; any modification in the ring structure leads to significant reduction of the enzymatic reaction [17]. The selectivity for the aromatic ring is stringent and UbiA and MenA enzymes do not prenylate DHNA and 4-hydroxybenzoate interchangeably even when both enzymes are active in the same bacterium as can been seen by the lack of menaquinone in the *E*. *coli* Δ*menA* strain (Fig 2). A similar stringency is also displayed by known chlorophyll and phylloquinone isoprenyltransferases [38,39].

The natural allylic isoprenyl diphosphate substrate for MenA in *M*. *tuberculosis* is solanesyl diphosphate, which has nine isoprene units (45 carbon atoms); however the enzyme is not as strict in its requirements for this substrate as it is for the aromatic substrate. FPP (three isoprene units, 15 carbon atoms) and GGPP (four isoprene units, 20 carbon atoms) were utilized with similar efficiency in preliminary experiments and an apparent K_m_ value of 4.3 μM was determined for FPP. This is in keeping with the previous observation that membrane preparations from *E*. *coli* and *M*. *luteus* can carry out this reaction in the presence of allylic isoprenyldiphosphates of varying chain lengths [15,17]. However, geranyl diphosphate (with two isoprene units, 10 carbon atoms) was not a substrate. Thus, *M*. *tuberculosis* MenA is capable of utilizing a variety of allylic isoprenyl diphosphates as substrates but has a requirement for at least three isoprene units.

Ro 48–8071 is a potent inhibitor of oxidosqualene cyclase and the squalene/hopane cyclase class of enzymes [40,41]. We have previously demonstrated that this compound inhibits the growth of *M*. *tuberculosis* even though this organism does not appear to synthesize cholesterol [42] or hopanes. These studies indicated that treatment with Ro 48–8071 and synthesized analogs inhibited MK synthesis and oxygen consumption in mycobacteria [3]. Complete inhibition of oxygen consumption was observed when Ro 48–8071 was added to cultures at 25 μg/ml. Moreover, the inhibition of oxygen consumption was reversible and was abolished by supplementing mycobacterial cultures with Vitamin K and analogs.

To further understand the mode of action of Ro 48–8071 on *M*. *tuberculosis* MenA activity, we examined the effects of Ro 48–8071 on the steady-state kinetics of the enzyme. The data indicates that Ro 48–8071 is a competitive inhibitor with respect to the allylic prenyl donor (FPP in this case); whereas, Ro 48–8071 is noncompetitive with respect to DHNA.

Thus, MenA catalyzes an interesting reaction that is poorly understood mechanistically, has not been purified and has significant implications as a potential drug target in pathogens that synthesize MK as their sole lipoquinone. Characterization of the activity has increased our understanding of the enzyme and should help facilitate design of inhibitors.

## Supporting information

S1 Table Strains, plasmids and primers used in these studies. (DOCX)

S1 Fig Effect of CHAPS concentration on MenA activity. Reaction mixtures contained 0.1 M Tris-HCl buffer (pH 8.0), 5 mM MgCl_2_, 2.5 mM dithiothreitol, membrane protein preparations (100 μg) from *M*. *tuberculosis*, DHNA (500 μM), [1-^3^H]farnesyl diphosphate (10 μM) and CHAPS at the indicated concentration. Mixtures were incubated at 37°C for 30 min and processed as described in Materials and Methods section. The inset shows the effect of various detergents on MenA activity when added to the reaction mixture at a final concentration of 0.5%.
(TIF)

S2 Fig Multiple sequence alignment of amino acid residues of MenA and UbiA homologs. Mtbmena—*Mycobacterium tuberculosis* MenA; EcolimenA—*Escherichia coli* MenA; AfUbia—*Archaeoglobus fulgidus* UbiA; Ecoliubia—*Escherichia coli* UbiA; ApUbia—*Aeropyrum pernix* UbiA.
(TIF)

S1 File Statistical analysis of data presented in Figs 3 and 4. (DOCX)
