# Supplementary material for: Characterization of MenA (isoprenyl diphosphate:1,4-dihydroxy-2-naphthoate isoprenyltransferase) from Mycobacterium tuberculosis
Source: PLoS One. 2019 Apr 12;14(4):e0214958. doi: 10.1371/journal.pone.0214958 (PMC6461227; doi:10.1371/journal.pone.0214958)
Supplement: S1 Table — (DOCX) [file pone.0214958.s001.docx]

**Table S1.** Strains, Plasmids and primers used in these studies.

**Strains**

*E. coli* Parent strain for the Keio collection of single-gene

(CGSC knockouts, *Δ(araD-araB)567*, *ΔlacZ4787*(::rrnB-3), *λ*^-^,

#7636) *rph-1*, *Δ(rhaD-rhaB)568*, *hsdR514* Ref 18

*E. coli* *E. coli* *ΔmenA* mutant,

(CGSC *Δ(araD-araB)567*, *ΔlacZ4787*(::rrnB-3), *λ*^-^, *rph-1*,

#10816) *Δ(rhaD-rhaB)568*, *ΔmenA789::*kan, *hsdR514* Ref 18

BL21(DE3) General purpose expression host, F^-^ *ompT hsdS_B_*(*r_B_^-^ m_B_^-^*)

*gal dcm* (DE3)

*E. coli* *E. coli* *ΔmenA* mutant complemented with pVV16:MenA

(CGSC

#10816) +

pVV16:MenA This study

BL21(DE3)+ Expression host transformed with expression vector

pET:MenA This study

**Plasmids**

pAPA3 derivative of pINT3, PacI site for cloning inserts,

Ag85a promoter and a single PacI site Ref 20

p2NIL cloning vector. *kan* Ref 19

pGOAL19 *hyg*, *lacZ*, *sacB* cassette, oriE, *amp* Ref 19

pUC-Hyg-Int integrating vector, *hyg*, L5 *int*, L5 attP Ref 23

pTACK102G suicide deletion vector, menAΔ, *hyg*, *lacZ*, *sacB, kan* This study

pTACK119 complementing vector, P_Ag85a_-*menA*, *gm*, L5 *int* This study

pVV16 *E. coli*-*Mycobacterium* shuttle and expression vector

used for constitutive protein expression from the

*hsp60* promoter, Kan^r^ and HygB^r^ Ref 24

pVV16:MenA pVV16 carrying *menA* between NdeI and HindIII sites

to allow the expression of MenA from *hsp60* promoter,

complementing vector This study

pET28a(+) Bacterial expression vector with T7lac promoter, adds

N-terminal His tag, thrombin cleavage site, internal T7

epitope, C-terminal His tag, Kan^r^

pET:MenA pET28a(+) carrying *menA* between NdeI and

HindIII sites, complementing vector This study

**Primers (restriction sites underlined)**

MenAF1 GCTGCAGTGACACACCAGATCGACACC

MenAR1 GAAGCTTGAGACCCACTGTGCGAAACT

MenAF2 GAACTTCATTGGCGTTTGGTCAGTT

MenAR2 CGGTACCCAACTGCAGGTTCTTGACCA

MenAF3 AATGATCATATGGCCAGTTTCGCACAGTGGGTC

MenAR3 AACAAGCTTGCCGCTCAACTGACCAAACGCCAATGC

MenAF4 TATATCATATGGCCAGTTTCGCACAGTGGGTC

MenAR4 TATATAAGCTTAGCTTAGCTCAACTGACCAAACGCCAATGC

MenAC1 GCGCCGAGTACTGGTGAT

MenAC2 TCTGTTCTCGTCGAGTGTGC
