# Supplementary figures and images for: Characterization of MenA (isoprenyl diphosphate:1,4-dihydroxy-2-naphthoate isoprenyltransferase) from Mycobacterium tuberculosis
Source: PLoS One. 2019 Apr 12;14(4):e0214958. doi: 10.1371/journal.pone.0214958 (PMC6461227; doi:10.1371/journal.pone.0214958)

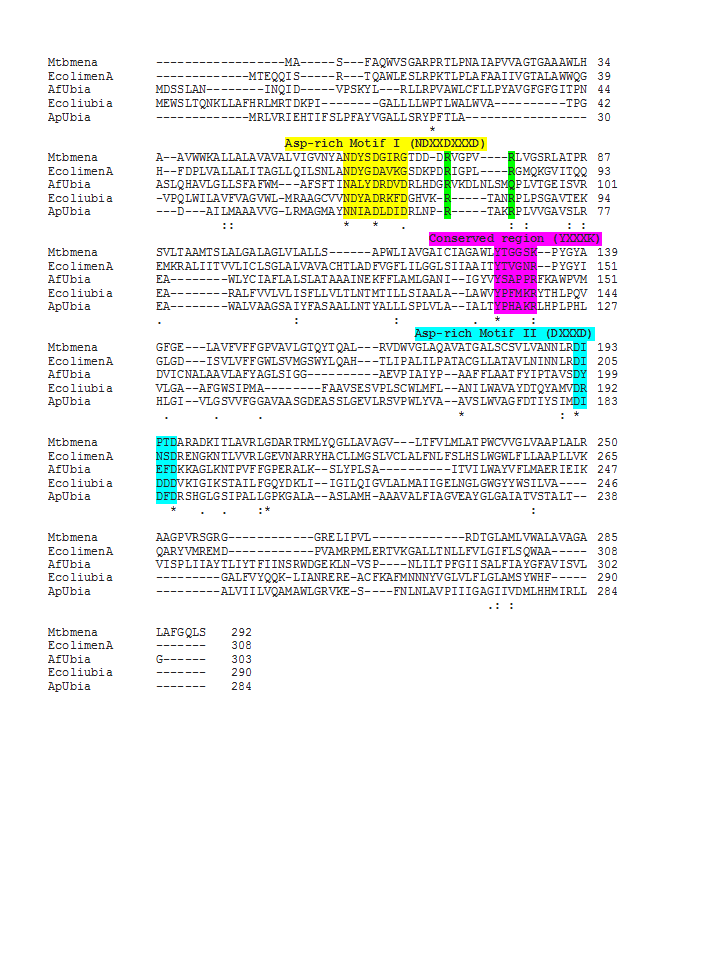

Supplement: S2 Fig — Mtbmena—Mycobacterium tuberculosis MenA; EcolimenA—Escherichia coli MenA; AfUbia—Archaeoglobus fulgidus UbiA; Ecoliubia—Escherichia coli UbiA; ApUbia—Aeropyrum pernix UbiA. (TIF) [file pone.0214958.s003.tif]
