# Supplementary material for: Characterization of MenA (isoprenyl diphosphate:1,4-dihydroxy-2-naphthoate isoprenyltransferase) from Mycobacterium tuberculosis
Source: PLoS One. 2019 Apr 12;14(4):e0214958. doi: 10.1371/journal.pone.0214958 (PMC6461227; doi:10.1371/journal.pone.0214958)
Supplement: S1 File — (DOCX) [file pone.0214958.s004.docx]

**Curve fit for Fig 3 [pET28a(+)]**

**Nonlinear Regression**

**Equation: Hyperbola, Single Rectangular, 2 Parameter**

f = a*x/(b+x)

**R Rsqr Adj Rsqr Standard Error of Estimate**

0.9977 0.9954 0.9946 0.0864

**Coefficient Std. Error t P**

a 16.9968 5.7774 2.9420 0.0259

b 126.0908 51.5665 2.4452 0.0501

**Analysis of Variance:**

**DF SS MS**

Regression 2 16.8937 8.4469

Residual 6 0.0447 0.0075

Total 8 16.9385 2.1173

Corrected for the mean of the observations:

**DF SS MS F P**

Regression 1 9.7060 9.7060 1301.4809 <0.0001

Residual 6 0.0447 0.0075

Total 7 9.7507 1.3930

**Statistical Tests:**

**Normality Test (Shapiro-Wilk)** Passed (P = 0.1252)

W Statistic= 0.8618 Significance Level = 0.0500

**Constant Variance Test** Failed (P = 0.0374)

**Fit Equation Description:**

[Variables]

x = col(1)

y = col(2)

reciprocal_y = 1/abs(y)

reciprocal_ysquare = 1/y^2

reciprocal_x = 1/abs(x)

reciprocal_xsquare = 1/x^2

reciprocal_pred = 1/abs(f)

reciprocal_predsqr = 1/f^2

weight_Cauchy = 1/(1+4*(y-f)^2)

[Parameters]

a = max(y) ''Auto {{previous: 16.9968}}

b = if(x50(x,y,0.1)<>0, x50(x,y,0.1), 1) ''Auto {{previous: 126.091}}

[Equation]

f = a*x/(b+x)

fit f to y

''fit f to y with weight reciprocal_y

''fit f to y with weight reciprocal_ysquare

''fit f to y with weight reciprocal_x

''fit f to y with weight reciprocal_xsquare

''fit f to y with weight reciprocal_pred

''fit f to y with weight reciprocal_predsqr

''fit f to y with weight weight_Cauchy

[Constraints]

[Options]

tolerance=1e-10

stepsize=1

iterations=200

Number of Iterations Performed = 12

**Curve fit for Fig 3 (pET28a:menA)**

**Nonlinear Regression**

**Equation: Hyperbola, Single Rectangular, 2 Parameter**

f = a*x/(b+x)

**R Rsqr Adj Rsqr Standard Error of Estimate**

0.9919 0.9839 0.9812 0.7372

**Coefficient Std. Error t P**

a 16.0936 0.8881 18.1217 <0.0001

b 4.0876 0.7043 5.8034 0.0011

**Analysis of Variance:**

**DF SS MS**

Regression 2 585.8174 292.9087

Residual 6 3.2606 0.5434

Total 8 589.0780 73.6347

Corrected for the mean of the observations:

**DF SS MS F P**

Regression 1 199.4071 199.4071 366.9442 <0.0001

Residual 6 3.2606 0.5434

Total 7 202.6677 28.9525

**Statistical Tests:**

**Normality Test (Shapiro-Wilk)** Passed (P = 0.5773)

W Statistic= 0.9365 Significance Level = 0.0500

**Constant Variance Test** Failed (P = 0.0212)

**Fit Equation Description:**

[Variables]

x = col(1)

y = col(3)

reciprocal_y = 1/abs(y)

reciprocal_ysquare = 1/y^2

reciprocal_x = 1/abs(x)

reciprocal_xsquare = 1/x^2

reciprocal_pred = 1/abs(f)

reciprocal_predsqr = 1/f^2

weight_Cauchy = 1/(1+4*(y-f)^2)

[Parameters]

a = max(y) ''Auto {{previous: 16.0936}}

b = if(x50(x,y,0.1)<>0, x50(x,y,0.1), 1) ''Auto {{previous: 4.08756}}

[Equation]

f = a*x/(b+x)

fit f to y

''fit f to y with weight reciprocal_y

''fit f to y with weight reciprocal_ysquare

''fit f to y with weight reciprocal_x

''fit f to y with weight reciprocal_xsquare

''fit f to y with weight reciprocal_pred

''fit f to y with weight reciprocal_predsqr

''fit f to y with weight weight_Cauchy

[Constraints]

[Options]

tolerance=1e-10

stepsize=1

iterations=200

Number of Iterations Performed = 7

**Curve fit for Fig 4 (Mg^++^ concentration)**

**Nonlinear Regression**

**Equation: Hyperbola, Single Rectangular, 2 Parameter**

f = a*x/(b+x)

**R Rsqr Adj Rsqr Standard Error of Estimate**

0.9542 0.9104 0.8925 0.0066

**Coefficient Std. Error t P**

a 0.0712 0.0045 15.6487 <0.0001

b 0.4897 0.1702 2.8782 0.0347

**Analysis of Variance:**

**DF SS MS**

Regression 2 0.0208 0.0104

Residual 5 0.0002 4.4035E-005

Total 7 0.0210 0.0030

Corrected for the mean of the observations:

**DF SS MS F P**

Regression 1 0.0022 0.0022 50.8159 0.0008

Residual 5 0.0002 4.4035E-005

Total 6 0.0025 0.0004

**Statistical Tests:**

**Normality Test (Shapiro-Wilk)** Failed (P = 0.0022)

W Statistic= 0.6785 Significance Level = 0.0500

**Constant Variance Test** Passed (P = 0.1209)

**Fit Equation Description:**

[Variables]

x = col(2)

y = col(3)

reciprocal_y = 1/abs(y)

reciprocal_ysquare = 1/y^2

reciprocal_x = 1/abs(x)

reciprocal_xsquare = 1/x^2

reciprocal_pred = 1/abs(f)

reciprocal_predsqr = 1/f^2

weight_Cauchy = 1/(1+4*(y-f)^2)

[Parameters]

a = max(y) ''Auto {{previous: 0.0711634}}

b = if(x50(x,y,0.1)<>0, x50(x,y,0.1), 1) ''Auto {{previous: 0.489733}}

[Equation]

f = a*x/(b+x)

fit f to y

''fit f to y with weight reciprocal_y

''fit f to y with weight reciprocal_ysquare

''fit f to y with weight reciprocal_x

''fit f to y with weight reciprocal_xsquare

''fit f to y with weight reciprocal_pred

''fit f to y with weight reciprocal_predsqr

''fit f to y with weight weight_Cauchy

[Constraints]

[Options]

tolerance=1e-10

stepsize=1

iterations=200

Number of Iterations Performed = 7
